# Maternal Vaccine Receipt and Infant Hospital and Emergency Visits for Influenza and Pertussis

**DOI:** 10.1001/jamanetworkopen.2025.53179

**Published:** 2026-01-08

**Authors:** Gabriella Morabito, Giovanni Corrao, Carlo Giaquinto, Anna Cantarutti, Costanza Di Chiara

**Affiliations:** 1 National Centre for Healthcare Research and Pharmacoepidemiology, at the University of Milano-Bicocca Milan, Italy; 2 Laboratory of Healthcare Research and Pharmacoepidemiology, Unit of Biostatistics, Epidemiology and Public Health, Department of Statistics and Quantitative Methods, University of Milano-Bicocca, Milan, Italy; 3 University of Milano-Bicocca (Emeritus Professor), Milan, Italy; 4 Department for Women’s and Children’s Health, University of Padua, Padua, Italy

## Abstract

**Importance:**

Influenza and tetanus-diphtheria-acellular pertussis (Tdap) vaccinations during pregnancy offer protection to infants from infections. However, evidence about their effectiveness against hospitalization and emergency department (ED) visits associated with influenza and pertussis remains limited.

**Objective:**

This study aimed to evaluate the association of maternal influenza and Tdap vaccinations with influenza- and pertussis-related hospitalizations and ED visits in infants younger than 6 months.

**Design, Setting, and Participants:**

This population-based cohort study used the health care utilization databases from the Lombardy region of Italy. Pregnant individuals who received the influenza and Tdap vaccine among all live-birth pregnancies in 2018 to 2022 were included. Each vaccinated mother was matched with a nonvaccinated counterpart based on month and year of delivery, gestational age at birth, and pregnancy multiplicity. Analyses were performed from April 2024 to February 2025.

**Exposures:**

Exposures of interest were influenza and Tdap vaccinations during pregnancy.

**Main Outcomes and Measures:**

The primary outcomes were infant hospitalizations or ED visits due to influenza and pertussis. Cox regression models were fitted to estimate the hazard ratio (HR) of each outcome associated with the corresponding maternal vaccine. Vaccine effectiveness (VE) was calculated as VE = (1 − HR) × 100%.

**Results:**

This study included 53 448 pregnant individuals who received the Tdap vaccine and 5347 who received influenza vaccine. The maternal vaccination coverage (ie, proportion of vaccinated pregnant individuals among those eligible) was 5359 (6.4%) for influenza and 70 119 (41.0%) for Tdap, respectively. Infants born to mothers who received the influenza and Tdap vaccine had a lower risk of hospitalization or ED visit for influenza (VE, 69.7%; 95% CI, 8.7%-90.0%) and pertussis (VE, 88.6%; 95% CI, 11.5%-98.5%), respectively.

**Conclusions and Relevance:**

This study found that maternal influenza and Tdap vaccinations were associated with reduced influenza- and pertussis-related hospitalization or ED visits in infants younger than 6 months. Given the low vaccination coverage, it is crucial to implement maternal vaccination campaigns to enhance infant health outcomes.

## Key Points

**Question:**

Are maternal influenza and Tdap vaccinations associated with influenza- and pertussis-related hospitalizations and emergency department (ED) visits in infants younger than 6 months?

**Findings:**

In this cohort study of 84 348 mother-infant dyads in the influenza cohort and 171 141 mother-infant dyads in the Tdap cohort, a strong negative association between maternal influenza and Tdap vaccinations and influenza- and pertussis-related hospitalization or ED visits in infants younger than 6 months was found, with an estimated vaccine effectiveness of 69.7% and 88.6%, respectively. Additionally, our findings confirm the suboptimal vaccine uptake in Italy.

**Meaning:**

These findings suggest support for the current recommendations for administering the Tdap and influenza vaccines during pregnancy and underline the urgent need to implement strategies to improve their acceptance.

## Teaser

This cohort study examined the association of influenza and tetanus-diphtheria-acellular pertussis vaccinations during pregnancy and infant hospitalization or emergency department visits in northern Italy.

## Introduction

Influenza and pertussis are major causes of morbidity and mortality worldwide, particularly among pregnant individuals and young infants, who are at higher risk of severe complications.^1,2^ Infants younger than 6 months are not eligible for influenza vaccination, and infants younger than 2 months are not eligible for diphtheria, tetanus toxoid, and acellular pertussis (Tdap) vaccinations.^3^ Therefore, maternal vaccination during pregnancy represents an effective strategy to confer passive protection to infants and mitigate the risk of infection and severe outcomes in this vulnerable population.^3,4,5^

Indeed, growing evidence supports the effectiveness of maternal vaccination in reducing the risk of infections in infants during their first months of life. Studies have documented that maternal influenza vaccination can reduce infant influenza incidence by 48% to 80%,^6,7,8,9,10^ while maternal pertussis vaccination has been associated with a 69% to 91% reduction in infant pertussis cases.^9,11^

However, systematic reviews have raised concerns regarding study design limitations, such as selection bias, heterogeneous case definitions, residual confounding, and the impact of seasonal influenza viral-vaccine mismatch, all of which may impact vaccine effectiveness (VE) estimates.^8,11^ Therefore, further observational evaluations are necessary to strengthen the evidence base. Moreover, while there is consistent evidence supporting this protective effect of maternal vaccination against infant influenza and pertussis,^6,7,9,10,11^ few studies have specifically explored its effectiveness in preventing infections requiring hospitalization or emergency department (ED) visits.^10^ To our knowledge, no studies of this nature have been conducted in Italy.

In Italy, the National Immunization Plan (2017 to 2019) of the Ministry of Health recommends and provides free maternal Tdap and influenza vaccination.^12^ The Tdap vaccine is ideally administered between the 27th and 36th gestational week, while influenza vaccination is available at any stage of pregnancy. However, Tdap and influenza vaccine uptake remains suboptimal in Italy as observed in other European countries.^6,13,14^ Addressing the existing evidence gap on VE in preventing influenza- and pertussis-related hospitalizations and ED visits could support public health efforts to improve maternal vaccine acceptance among pregnancy individuals and health care professionals. This study aimed to evaluate the association of maternal influenza and Tdap vaccinations in pregnancy with infections requiring hospitalization or ED visits in infants younger than 6 months.

## Methods

### Setting

This study used data from the health care utilization databases of Lombardy, a northern region of Italy, that accounts for approximately 16% (nearly 10 million) of the national population, all of whom are covered by the National Health Service (NHS). Each region in Italy maintains an automated database system that collects comprehensive information on the provided health services free of charge, including (1) demographic and administrative data of NHS beneficiaries, including the start and end dates of coverage by the NHS; (2) hospital discharge records, with diagnosis and procedures coded according to the *International Classification of Diseases, Ninth Revision, Clinical Modification *(*ICD-9-CM*); (3) outpatient drug dispensing, coded using the Anatomical Therapeutic Chemical (ATC) classification system; (4) vaccination registry; (5) copayment exception for chronic disease; and (6) Certificates of Delivery Assistance (CeDAP), which contain detailed socioeconomic and medical information on pregnancy, childbirth, and newborn presentation at delivery. Additional details on the health care utilization databases of the Lombardy region in the field of pregnancy and child health are available elsewhere.^15,16^ These databases are linked through a unique identification code assigned at birth, enabling the creation of an extensive and unselected maternal-birth cohort and allowing for the longitudinal tracking of care pathways for pregnant individuals and newborns. To ensure privacy, each identification code is automatically deidentified by the Lombardy region, with the inverse process only being allowed by the Regional Health Authority on request from judicial authorities.

According to the rules of the Italian Medicines Agency,^17^ retrospective studies using administrative databases do not require protocol approval by Ethics Committees. Informed consent was waived due to the retrospective nature of the study. We followed the Strengthening the Reporting of Observational Studies in Epidemiology (STROBE) reporting guideline for this cohort study.^18^

### Cohorts Selection

Using the CeDAP database, we identified all live-birth pregnancies between January 1, 2018, and November 30, 2022, among pregnant individuals aged 12 to 55 years at delivery with gestational periods ranging from 22 to 42 weeks, based on the date of the last menstrual period (LMP) ascertained through maternal report or ultrasonography. Pregnancies were excluded if pregnant individuals (1) did not have continuous NHS enrollment from 3 months before the LMP until 1 month after delivery; (2) lacked a hospital admission *ICD-9-CM* code for delivery; (3) could not be linked to their newborns due to a missing identification code; or (4) had missing sociodemographic information (approximately 0.1% of the total). eTable 1 in Supplement 1 reports the comparison between the characteristics of included dyads and those excluded due to lack of an identification code. With regard to influenza, only deliveries occurring between September 1 and February 28 were included to ensure that infants’ early months of life coincided with the influenza season. Two separate cohorts of mother-infant dyads were identified for influenza and Tdap vaccinations, respectively.

### Maternal Influenza and Tdap Vaccination During Pregnancy

For each mother-infant dyad, maternal vaccination status was determined based on vaccine administration records during pregnancy. Infants whose mothers had received the influenza or Tdap vaccine anytime from the LMP to delivery were classified as exposed to the corresponding vaccine.

### Matching Design

Each vaccinated mother-infant dyad (influenza or Tdap) was matched to 1 unvaccinated dyad from the same vaccine group. Matching was performed on month and year of delivery, gestational age at birth, and pregnancy multiplicity. Infants were followed from birth until the earliest of the following events: 6 months of age, clinical outcome (defined below as hospitalization or ED visits for influenza or pertussis, respectively), emigration, death, or end of the study period (December 31, 2022). In the Tdap cohort, as pertussis vaccination is mandatory in Italy and generally administered at 8 weeks of age, follow-up was additionally censored at the vaccination date, if available. Overall, 96% of infants received vaccination by the end of follow-up.

### Infant Outcome

The primary outcome of interest was a composite endpoint of hospital admission or ED visits with a diagnosis of influenza (code: 487) and pertussis (*ICD-9-CM* codes: 033; 484.3) within 6 months post-birth. The date of the earliest hospitalization or ED visit was recorded as the onset time of each clinical outcome.

### Covariates

A set of other potential confounders was considered, including maternal sociodemographic and clinical characteristics. There included maternal employment status (employed vs unemployed), education level (categorized as low, none or primary education; intermediate, secondary education; and high, high school or higher), marital status (married vs unmarried), parity (nulliparous vs multiparous), and chronic disease status. Chronic diseases were identified based on active copayment exceptions for conditions, such as asthma, diabetes, chronic obstructive pulmonary disease, HIV infection, chronic respiratory failure, lupus, multiple sclerosis, and malignancies at the time of the LMP.

### Statistical Analysis

Standardized mean differences (SMDs) were used to compare differences between exposed and unexposed groups. Covariate balance was considered achieved when the absolute SMD between groups was less than 0.1.^19^ Vaccination coverage was calculated as the ratio between the number of pregnant individuals who received each vaccine and the total number of pregnant individuals eligible for vaccination (ie, those who delivered during the period of interest).

Adjusted cause-specific hazard regression models were implemented to estimate the hazard ratio (HR), and 95% CI, for the association between influenza or Tdap vaccinations during pregnancy and the outcome, accounting for death as competing event.^20^ The proportional hazards assumption was tested using the log-minus-log plot and the cumulative martingale-based residuals.^21^ VE was calculated as VE = (1 − HR) × 100%.

Given that the observation period includes the COVID-19 pandemic, which may have influenced both maternal vaccination decisions and infant susceptibility to influenza or pertussis, the models were also adjusted for the COVID-19 pandemic era (defined as starting from March 1, 2020), included as a time-dependent variable. To further address the potential impact of the COVID-19 pandemic, a sensitivity analysis was conducted by restricting the study population to infants born before February 1, 2020, and censoring their follow-up at March 1, 2020, thereby focusing on the prepandemic era.

Additionally, we performed 2 sensitivity analyses to account for potential nonadministrative censoring associated with infant pertussis vaccination—inverse probability of censoring weighting (IPCW) and the inclusion of an interaction between maternal Tdap vaccination status and time-dependent infant vaccination. Finally, a Poisson regression model was fitted as a sensitivity analysis to account for multiple ED visits or hospitalizations.

SAS version 9.4 (SAS Institute) was used for the analyses. For all hypotheses tested, 2-tailed *P* < .05 were considered to be significant. Data were analyzed from April 2024 to February 2025.

## Results

### Study Cohorts

Between January 2018 and November 2022, a total of 339 021 pregnancies were recorded. Of these, 84 348 mother-infant dyads met the inclusion and exclusion criteria for the influenza cohort (Figure). Among them, 5359 (6.4%) received the influenza vaccine in pregnancy, with 5166 (96.4%) vaccinated in the third trimester. A total of 171 141 mother-infant dyads were eligible for the Tdap cohort, of whom 70 119 (41.0%) received the Tdap vaccine, predominantly during the third trimester of pregnancy.

**Figure.**
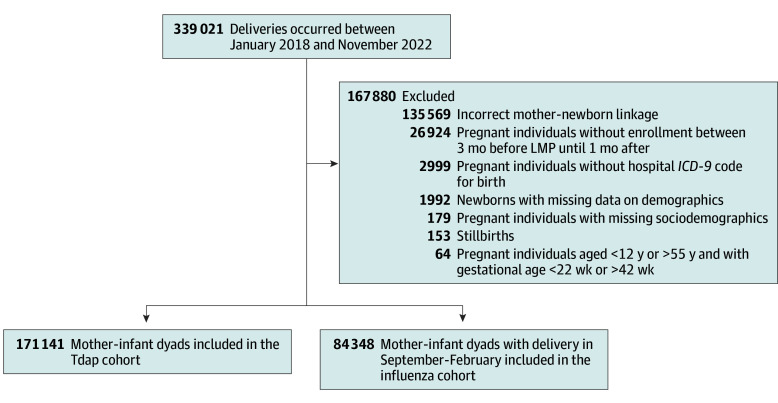
Flow Chart of Exclusion and Inclusion Criteria *ICD-9* indicates *International Classification of Diseases, Ninth Revision*; LMP, last menstrual period; Tdap, tetanus-diphtheria-acellular pertussis.

Successful matching was achieved for 5347 of 5359 influenza-vaccinated pregnant individuals (99.8%) and 53 448 of 70 119 Tdap-vaccinated pregnant individuals (76.2%), each matched with as many unvaccinated counterparts. Baseline characteristics of the unmatched cohorts are reported in eTables 2 and 3 in Supplement 1.

Table 1 and Table 2 show baseline characteristics of the matched cohorts. In the influenza cohort, vaccinated mothers were more often of Italian nationality (4489 [84.0%] vs 3924 [73.4%]) and employed (4133 [77.3%] vs 3694 [69.1%]), compared with their unvaccinated counterparts. Vaccinated mothers also had higher education levels (2680 [50.1%] vs 2217 [41.5%] with high school or higher), and were more frequently aged 25 to 34 years (2779 [52.0%]), with a slightly higher mean age compared with unvaccinated mothers. In the Tdap cohort, similar patterns were observed. A greater proportion of vaccinated mothers were Italian (42 981 [80.4%] vs 37 045 [69.3%]), employed (40 813 [76.4%] vs 34 577 [64.7%]), and had completed at least high school (24 833 [46.5%] vs 19 733 [36.9%]), compared with unvaccinated mothers. They were also more often aged 25 to 34 years (55.7%) and nulliparous (28 883 [56.3%] vs 22 414 [43.9%]).

**Table 1.** Characteristics of Pregnant Individuals Vaccinated With Influenza Vaccination During Pregnancy and of the Matched Individuals Who Did Not Receive the Vaccine

Characteristic	Influenza vaccination, No. (%)		SMD
	Unexposed (n = 5347)	Exposed (n = 5347)	
Gestational age, wk			
<37	371 (6.9)	371 (6.9)	MV
≥37	4976 (93.1)	4976 (93.1)	
Multiple delivery			
Yes	109 (2.0)	109 (2.0)	MV
No	5238 (98.0)	5238 (98.0)	
Age at delivery, y			
<25	325 (6.1)	218 (4.1)	0.112
25-34	2872 (53.7)	2779 (52.0)	
≥35	2150 (40.2)	2350 (44.0)	
Nationality			
Italian	3924 (73.4)	4489 (84.0)	0.260
Other	1423 (26.6)	858 (16.1)	
Marital status			
Married	3174 (59.4)	3279 (61.3)	0.040
Unmarried	2173 (40.6)	2068 (38.7)	
Education			
Low	985 (18.4)	677 (12.7)	0.208
Intermediate	2145 (40.1)	1990 (37.2)	
High	2217 (41.5)	2680 (50.1)	
Employment			
Employed	3694 (69.1)	4133 (77.3)	0.186
Unemployed	1653 (30.9)	1214 (22.7)	
Parity			
Nulliparous	2516 (47.1)	2732 (51.1)	0.081
Multiparous	2831 (53.0)	2615 (48.9)	
Infant’s sex			
Male	2745 (51.3)	2749 (51.4)	0.002
Female	2602 (48.7)	2598 (48.6)	
Chronic diseases			
Yes	182 (3.4)	234 (4.4)	0.050
No	5165 (96.6)	5113 (95.6)	

Abbreviations: MV, matching variable; SMD, standardized mean difference.

**Table 2.** Characteristics of Pregnant Individuals Vaccinated With Tdap Vaccination During Pregnancy and of the Matched Individuals Who Did Not Receive the Vaccine

Characteristic	Tdap vaccination, No. (%)		SMD
	Unexposed (n = 53 448)	Exposed (n = 53 448)	
Gestational age, wk			
<37	2832 (5.3)	2832 (5.3)	MV
≥37	50 616 (94.7)	50 616 (94.7)	
Multiple delivery			
Yes	615 (1.2)	615 (1.2)	MV
No	52 833 (98.9)	52 833 (98.9)	
Age at delivery, y			
<25	4124 (7.7)	2605 (4.9)	0.125
25-34	28 273 (52.9)	29 748 (55.7)	
≥35	21 051 (39.4)	21 095 (39.5)	
Nationality			
Italian	37 045 (69.3)	42 981 (80.4)	0.258
Other	16 403 (30.7)	10 467 (19.6)	
Marital status			
Married	31 991 (59.9)	31 606 (59.1)	0.015
Unmarried	21 457 (40.2)	21 842 (40.9)	
Education			
Low	11 743 (22.0)	7638 (14.3)	0.218
Intermediate	21 972 (41.1)	20 977 (39.3)	
High	19 733 (36.9)	24 833 (46.5)	
Employment			
Employed	34 577 (64.7)	40 813 (76.4)	0.258
Unemployed	18 871 (35.3)	12 635 (23.6)	
Parity			
Nulliparous	22 414 (43.9)	28 883 (56.3)	0.244
Multiparous	31 034 (56.1)	24 565 (46.0)	
Infant’s sex			
Male	27 522 (51.5)	27 387 (51.2)	0.005
Female	25 926 (48.5)	26 061 (48.8)	
Chronic diseases			
Yes	1708 (3.2)	1905 (3.6)	0.020
No	51 740 (96.8)	51 543 (96.4)	

Abbreviations: MV, matching variable; SMD, standardized mean difference; Tdap, tetanus-diphtheria-acellular pertussis.

### Infant Outcomes

The incidence of severe influenza and pertussis was 392 and 51 cases per 100 000 person-years, respectively. Crude and adjusted hazard ratios for the association between maternal vaccination and infant outcomes are reported in Table 3. Infants born to mothers vaccinated against influenza during pregnancy had a significantly lower risk of hospitalization or ED visit for influenza (VE, 69.7%; 95% CI, 8.7%-90.0%; HR, 0.30; 95% CI, 0.10-0.91). Similarly, maternal Tdap vaccination was associated with a reduced risk of hospitalization or ED visit for pertussis (VE, 88.6%; 95% CI, 11.5%-98.5%; HR, 0.11; 95% CI, 0.02-0.88). Sensitivity analysis restricted to the prepandemic era confirmed a lower risk for both influenza (HR, 0.13; 95% CI, 0.02-0.1.04) and pertussis (HR, 0.13; 95% CI, 0.02-0.99) in infants born to vaccinated mothers. The results were consistent when applying the IPCW and including the interaction term between maternal Tdap vaccination and time-dependent infant vaccination (eTable 4 in Supplement 1) and when fitting the Poisson regression model (eTable 5 in Supplement 1).

**Table 3.** Association Between Influenza and Pertussis Hospitalizations and ED Visits Among Infants Younger Than 6 Months and Maternal Influenza and Tdap Vaccine

Exposure	HR (95% CI)
**Main analysis**	
Crude	
Influenza vaccine	0.25 (0.08-0.75)
Tdap vaccine	0.09 (0.01-0.72)
Adjusted	
Influenza vaccine	0.30 (0.10-0.91)
Tdap vaccine	0.11 (0.02-0.88)
**Prepandemic era**	
Crude	
Influenza vaccine	0.11 (0.01-0.87)
Tdap vaccine	0.10 (0.01-0.80)
Adjusted	
Influenza vaccine	0.13 (0.02-0.99)
Tdap vaccine	0.13 (0.02-1.04)

Abbreviations: ED, emergency department; HR, hazard ratio; Tdap, tetanus-diphtheria-acellular pertussis.

## Discussion

Our study provided further evidence of the negative association of maternal influenza and Tdap vaccinations with severe outcomes in infants, specifically 70% and 89% reductions in hospitalization and ED visits for influenza and pertussis, respectively. Our results aligned with studies conducted in the US and Australia that have documented a protective association between maternal vaccination and severe diseases in young infants.^9,10,22,24,25^ In particular, the maternal influenza vaccine has been shown to be associated with protection ranging from 39% to 92% against influenza-related hospitalizations in infants younger than 6 months,^10,22,23,24^ with only 1 study including data from the COVID-19 pandemic era.^10^ Similarly, Tdap VE in preventing pertussis-related hospitalization in infants younger than 6 months of age has been reported at 75% and 94%, respectively.^25,26^ By extending prior evidence, this study provided novel insights and, to our knowledge, represents the first investigation of this kind in the Italian setting.

Two additional key findings warrant attention. First, despite established recommendations, maternal vaccination coverage remained suboptimal, especially for influenza vaccination. In this context, providing evidence about vaccine effectiveness and increasing public awareness were particularly important. Second, as previously reported,^13,27^ vaccination acceptance was influenced by maternal sociodemographic factors, including higher education, employment status, and nationality. This highlighted the importance of implementing targeted public health strategies to improve vaccine uptake, particularly among vulnerable populations who may be less likely to receive vaccinations.

A major strength of our study was its use of comprehensive health care use databases, alongside the CeDAP registry in Lombardy, which enabled a large, population-based cohort study. This was facilitated by Italy’s universal health care system, which ensured near-complete data capture. Moreover, because health care utilization data were collected for administrative purposes, biases, such as recall bias, nonresponse bias, and loss to follow-up, were inherently minimized.

### Limitations

This study has limitations. First, our data include only health services and vaccine administrations within the NHS reimbursement system and exclude those provided at the private practice level. However, exposure misclassification is unlikely, as vaccines administered outside the NHS are known to constitute less than 9% of the total,^28^ and, given their free-of-charge availability of influenza and Tdap vaccines during pregnancy, this proportion is likely even lower. Moreover, since the private vaccination decision is usually driven by logistical or personal preferences rather than by infants’ subsequent risk of severe infections, any misclassification is plausibly nondifferential and would bias results toward the null, likely underestimating the true protection associated with vaccination. Second, some outcome misclassification may also be present, as we were unable to verify the accuracy of hospital diagnoses. Third, due to limited statistical power, we were unable to conduct subgroup analyses based on gestational age or the trimester in which each vaccine was administered. Finally, given the observational nature of our study and the absence of data on lifestyle factors, such as alcohol use, smoking, or health-seeking behaviors, residual confounding due to behavioral differences cannot be fully excluded.

## Conclusions

Our study revealed a strong association between maternal influenza and Tdap vaccinations and reduced influenza- and pertussis-related hospitalizations and ED visits in infants younger than 6 months of age, alongside evidence of suboptimal vaccine uptake during pregnancy. These results support the current recommendations for administering these vaccines during pregnancy and highlight the urgent need to implement strategies aimed to increase their acceptance.

## Notes

Supplement 1. **eTable 1. **Characteristics of Linked Mother-Infant Dyads Compared With Those Without Identification Code (Excluded) **eTable 2. **Baseline Characteristics Before Matching: Influenza-Vaccinated vs Unvaccinated Pregnant Individuals **eTable 3.** Baseline Characteristics Before Matching: Tdap-Vaccinated vs Unvaccinated Pregnant Individuals **eTable 4. **aHRs and 95% CIs Estimating the Association Between Pertussis Hospitalizations/ED Visits and Maternal Tdap Vaccine, Using IPCW and the Interaction With Time-Dependent Infant Vaccination **eTable 5. **Adjusted IRR and 95% CIs Estimating the Association Between Pertussis Hospitalizations/ED Visits and Maternal Influenza and Tdap Vaccine

Supplement 2.
Data Sharing Statement
