# Supplementary material for: Maternal Vaccine Receipt and Infant Hospital and Emergency Visits for Influenza and Pertussis
Source: JAMA Netw Open. 2026 Jan 8;9(1):e2553179. doi: 10.1001/jamanetworkopen.2025.53179 (PMC12784226; doi:10.1001/jamanetworkopen.2025.53179)
Supplement: Supplement 1. — eTable 1. Characteristics of Linked Mother-Infant Dyads Compared With Those Without Identification Code (Excluded) eTable 2. Baseline Characteristics Before Matching: Influenza-Vaccinated vs Unvaccinated Pregnant Individuals eTable 3. Baseline Characteristics Before Matching: Tdap-Vaccinated vs Unvaccinated Pregnant Individuals eTable 4. aHRs and 95% CIs Estimating the Association Between Pertussis Hospitalizations/ED Visits and Maternal Tdap Vaccine, Using IPCW and the Interaction With Time-Dependent Infant Vaccination eTable 5. Adjusted IRR and 95% CIs Estimating the Association Between Pertussis Hospitalizations/ED Visits and Maternal Influenza and Tdap Vaccine [file jamanetwopen-e2553179-s001.pdf]

Supplemental Online Content

Morabito G, Corrao G, Giaquinto C, Cantarutti A, Di Chiara C. Maternal vaccine effectiveness on infant hospital and emergency visits for influenza and pertussis. *JAMA Netw Open*. 2026;9(1):e2553179. doi:10.1001/jamanetworkopen.2025.53179

eTable 1. Characteristics of Linked Mother-Infant Dyads Compared With Those Without Identification Code (Excluded)

eTable 2. Baseline Characteristics Before Matching: Influenza-Vaccinated Vs Unvaccinated Pregnant Individuals

eTable 3. Baseline Characteristics Before Matching: Tdap-Vaccinated Vs Unvaccinated Pregnant Individuals

eTable 4. aHRs and 95% CIs Estimating the Association Between Pertussis Hospitalizations/ER Visits and Maternal Tdap Vaccine, Using IPCW and the Interaction With Time-Dependent Infant Vaccination

eTable 5. Adjusted IRR and 95% CIs Estimating the Association Between Pertussis Hospitalizations/ER Visits and Maternal Influenza and Tdap Vaccine

This supplemental material has been provided by the authors to give readers additional information about their work.

© 2026 Morabito et al. *JAMA Network Open*.

eTable 1. Characteristics of Linked Mother-Infant Dyads Compared With Those Without Identification Code (Excluded)

	Correct mother-infant linkage (171,141)	Incorrect mother-infant linkage (132,135)	Standardized mean differences
Gestational age			0.020
< 37 weeks	10,659 (6.2%)	7,604 (5.8%)	
≥ 37 weeks	160,482 (93.8%)	124,531 (94.2%)	
Age at delivery			0.086
< 25 years	10,943 (6.4%)	9,026 (6.8%)	
25–34 years	93,406 (54.6%)	76,445 (57.9%)	
≥ 35 years	66,792 (39.0%)	46,664 (35.3%)	
Nationality			0.013
Italian	128,091 (74.9%)	99,616 (75.4%)	
Other	43,050 (25.1%)	32,519 (24.6%)	
Marital status			0.013
Married	102,651 (60.0%)	78,408 (59.3%)	
Unmarried	68,490 (40.0%)	53,727 (40.7%)	
Education			0.110
Low	69,952 (40.9%)	47,682 (36.1%)	
Intermediate	69,483 (40.6%)	56,666 (42.9%)	
High	31,706 (18.5%)	27,787 (21.0%)	
Employment			0.043
Employed	120,583 (70.5%)	90,491 (68.5%)	
Unemployed	50,558 (29.6%)	41,644 (31.5%)	
Parity			0.032
Nulliparous	82,584 (48.4%)	61,868 (46.8%)	
Multiparous	88,287 (54.6%)	70,267 (53.2%)	
5-minute Apgar score			0.013
<7	1,035 (0.6%)	937 (0.7%)	
7-10	170,106 (99.4%)	131,198 (99.3%)	
Low Birth Weight			0.012
Yes	10,388 (6.1%)	7,638 (5.8%)	
No	160,753 (93.9%)	124,497 (94.2%)	

© 2026 Morabito et al. *JAMA Network Open*.

eTable 2. Baseline Characteristics Before Matching: Influenza-Vaccinated vs Unvaccinated Pregnant Individuals

	Influenza vaccination		Standardized mean differences
	**Unexposed (N=78,989)**	**Exposed (N=5,359)**	
Gestational age			0.039
< 37 weeks	74,104 (93.8%)	4,976 (92.8%)	
≥ 37 weeks	4,885 (6.2%)	383 (7.2%)	
Multiple delivery			0.000
Yes	1,283 (1.6%)	116 (2.2%)	
No	77,706 (98.4%)	5,243 (97.8%)	
Age at delivery			0.126
< 25 years	5,133 (6.5%)	218 (4.1%)	
25–34 years	43,172 (54.7%)	2,787 (52.0%)	
≥ 35 years	30,684 (38.9%)	2,354 (43.9%)	
Nationality			0.237
Italian	58,737 (74.4%)	4,497 (83.9%)	
Other	20,252 (25.6%)	862 (16.1%)	
Marital status			0.039
Married	46,972 (59.5%)	3,288 (61.4%)	
Unmarried	32,017 (40.5%)	2,071 (38.6%)	
Education			0.222
Low	15,114 (19.1%)	678 (12.7%)	
Intermediate	32,219 (40.8%)	1,996 (37.3%)	
High	31,658 (40.1%)	2,685 (50.1%)	
Employment			0.167
Employed	55,270 (70.0%)	4,143 (77.3%)	
Unemployed	23,719 (30.0%)	1,210 (22.7%)	
Parity			0.052
Nulliparous	38,315 (48.5%)	2,739 (51.1%)	
Multiparous	40,674 (51.5%)	2,620 (48.9%)	
Infant's sex			0.003
Male	40,492 (51.3%)	2,754 (51.4%)	
Female	38,497 (48.8%)	2,605 (48.6%)	
Chronic diseases			0.063
Yes	2,509 (3.2%)	235 (95.6%)	
No	78,480 (96.8%)	5,124 (4.4%)	

© 2026 Morabito et al. *JAMA Network Open*.

eTable 3. Baseline Characteristics Before Matching: Tdap-Vaccinated vs Unvaccinated Pregnant Individuals

	Tdap vaccination		Standardized mean differences
	**Unexposed (N=101,022)**	**Exposed (N=70,119)**	
Gestational age			0.072
< 37 weeks	7,002 (6.9%)	3,657 (5.2%)	
≥ 37 weeks	94,020 (93.1%)	66,462 (94.8%)	
Multiple delivery			0.082
Yes	1,789 (1.8%)	1,051 (1.5%)	
No	999,233 (98.2%)	69,068 (98.5%)	
Age at delivery			0.122
< 25 years	7,808 (7.5%)	3,335 (4.8%)	
25–34 years	54,265 (53.7%)	30,141 (55.8%)	
≥ 35 years	39,149 (38.8%)	27,643 (39.4%)	
Nationality			0.231
Italian	71,535 (70.8%)	58,558 (80.7%)	
Other	20,487 (29.2%)	13,563 (19.3%)	
Marital status			0.050
Married	61,610 (61.0%)	41,041 (58.5%)	
Unmarried	39,412 (39.0%)	29,078 (41.5%)	
Education			0.234
Low	21,887 (21.1%)	9,819 (14.0%)	
Intermediate	42,140 (41.7%)	27,343 (39.0%)	
High	36,995 (36.6%)	32,857 (47.0%)	
Employment			0.240
Employed	66,717 (66.0%)	53,866 (76.8%)	
Unemployed	34,305 (34.0%)	16,253 (23.2%)	
Parity			0.201
Nulliparous	44,762 (44.3%)	38,092 (54.3%)	
Multiparous	56,260 (55.7%)	32,027 (45.7%)	
Infant’s sex			0.004
Male	52,053 (51.5%)	35,988 (51.3%)	
Female	48,969 (48.5%)	34,131 (48.7%)	
Chronic diseases			0.030
Yes	3,098 (3.1%)	2,524 (3.6%)	
No	97,924 (96.9%)	67,595 (96.4%)	

© 2026 Morabito et al. *JAMA Network Open*.

eTable 4. aHRs and 95% CIs Estimating the Association Between Pertussis Hospitalizations/ER Visits and Maternal Tdap Vaccine, Using IPCW and the Interaction with Time-Dependent Infant Vaccination

	**HR**	**95% CI**
IPCW	0·12	0·02–0·92
Interaction with infant vaccination	0·12	0·02–0·93

© 2026 Morabito et al. *JAMA Network Open*.

eTable 5. Adjusted IRR and 95% CIs Estimating the Association Between Pertussis Hospitalizations/ER Visits and Maternal Influenza and Tdap Vaccine

	**IRR**	**95% CI**
Influenza	0·28	0·13–0·60
Tdap	0·14	0·04–0·60

© 2026 Morabito et al. *JAMA Network Open*.
