# Supplementary material for: Maternal Vaccine Receipt and Infant Hospital and Emergency Visits for Influenza and Pertussis
Source: JAMA Netw Open. 2026 Jan 8;9(1):e2553179. doi: 10.1001/jamanetworkopen.2025.53179 (PMC12784226; doi:10.1001/jamanetworkopen.2025.53179)
Supplement: Supplement 2. — Data Sharing Statement [file jamanetwopen-e2553179-s002.pdf]

Data Sharing Statement

Morabito. Maternal Vaccine Receipt and Infant Hospital and Emergency Visits for Influenza and Pertussis. *JAMA Netw Open*. Published January 08, 2026.
doi:10.1001/jamanetworkopen.2025.53179

Data
Data available: No

Additional Information
Explanation for why data not available: The data that support the findings of this study are available from Lombardy Region but restrictions apply to the availability of these data, which were used under license for the current study, and so are not publicly available. Data are however available upon reasonable request and with permission of Lombardy Region. Requests to access these datasets should be directed to DG Welfare Lombardy Region, Epidemiology Observatory Organisational Unit (olivia_leoni@regione.lombardia.it).
